# Bacterial Diversity and Bioremediation Potential of the Highly Contaminated Marine Sediments at El-Max District (Egypt, Mediterranean Sea)

**DOI:** 10.1155/2015/981829

**Published:** 2015-07-27

**Authors:** Ranya A. Amer, Francesca Mapelli, Hamada M. El Gendi, Marta Barbato, Doaa A. Goda, Anna Corsini, Lucia Cavalca, Marco Fusi, Sara Borin, Daniele Daffonchio, Yasser R. Abdel-Fattah

**Affiliations:** ^1^Environmental Biotechnology Department, Genetic Engineering and Biotechnology Research Institute, City of Scientific Research and Technology Applications, Alexandria, Egypt; ^2^Department of Food, Environment and Nutritional Sciences (DeFENS), University of Milan, 20133 Milan, Italy; ^3^Bioprocess Development Department, Genetic Engineering and Biotechnology Research Institute, City of Scientific Research and Technology Applications, Alexandria, Egypt; ^4^Biological and Environmental Sciences and Engineering Division (BESE), King Abdullah University of Science and Technology (KAUST), Thuwal 23955, Saudi Arabia; ^5^Genetic Engineering and Biotechnology Research Institute (GEBRI), City for Scientific Research and Technology Applications (SRTA City), New Burg El-Arab City, Universities and Research Institutes District, Alexandria 21934, Egypt

## Abstract

Coastal environments worldwide are threatened by the effects of pollution, a risk particularly high in semienclosed basins like the Mediterranean Sea that is poorly studied from bioremediation potential perspective especially in the Southern coast. Here, we investigated the physical, chemical, and microbiological features of hydrocarbon and heavy metals contaminated sediments collected at El-Max bay (Egypt). Molecular and statistical approaches assessing the structure of the sediment-dwelling bacterial communities showed correlations between the composition of bacterial assemblages and the associated environmental parameters. Fifty strains were isolated on mineral media supplemented by 1% crude oil and identified as a diverse range of hydrocarbon-degrading bacteria involved in different successional stages of biodegradation. We screened the collection for biotechnological potential studying biosurfactant production, biofilm formation, and the capability to utilize different hydrocarbons. Some strains were able to grow on multiple hydrocarbons as unique carbon source and presented biosurfactant-like activities and/or capacity to form biofilm and owned genes involved in different detoxification/degradation processes. El-Max sediments represent a promising reservoir of novel bacterial strains adapted to high hydrocarbon contamination loads. The potential of the strains for exploitation for *in situ* intervention to combat pollution in coastal areas is discussed.

## 1. Introduction

The Mediterranean Sea is exposed to a high risk of pollution by petroleum hydrocarbons (HC), due to the presence of tens of sites related to their extraction, refinery, and transport along its coastline [1]. This risk is exacerbated by several factors, including the semienclosed nature of this sea and the geographical location of most of the oil-producing and oil-consuming countries, placed, respectively, on the Southern and Northern sides of the basin, entailing the presence of pipeline terminal and oil tanker traffic. A recent analysis of the papers published in the last years about the microbiology of coastal and open-sea sites in the Mediterranean Sea clearly showed that the Southern side of the basin has been largely neglected [2] although it hosts several polluted areas along its coasts, such as El-Max district area (Alexandria, Egypt). Due to the numerous industrial activities, the disposal of untreated waste effluents, and the shipping activities, El-Max bay is a coastal site chronically contaminated by crude oil and heavy metals [3] whose clean-up represents a challenge for the Egyptian country and for the entire research community. Crude oil is a mixture of organic compounds that may contain up to 20000 chemicals and it is hardly removable from polluted ecosystems by traditional methods [4]. Bioremediation is an alternative to physical and chemical methods and takes advantage of the natural ability of certain microbes to degrade HC, buffering the effect of oil pollution in natural ecosystems. Bioremediation can be achieved by adding nutrients to the autochthonous biodegrading microbes (biostimulation) or adding a microorganism's inoculum in the polluted environment (bioaugmentation). The successfulness of such approaches is still under debate [5–7]; however recent reports suggest the use of autochthonous bioaugmentation (ABA) as the best practice to restore polluted marine ecosystems [8]. The starting point for such approach is the detailed study of the diversity of microbial communities colonizing the polluted site of interest. Such survey should be accomplished through both molecular and cultivation dependent techniques that, respectively, allow (i) the correlation of the environmental parameters with the structure of the whole microbial communities and (ii) the enrichment, identification, and characterization of degrading microbes for traits of interest like the production of biosurfactant. Biosurfactants are molecules that have hydrophilic and hydrophobic moieties and, enhancing the bioavailability of oil hydrocarbons, are pivotal in microbial oil degradation network [8]. In this pipeline, the most promising microbes can be selected for subsequent laboratory scale experiments to test their degrading capability before* ex situ* and* in situ* field ABA trials.

This work represents the first holistic investigation of the bacterial communities inhabiting the marine sediments of different stations located in El-Max district bay. It aims to unravel the pattern of bacterial diversity, ecology, and degradation potential in polluted sediments and to obtain promising bacterial resources to be exploited for marine sites' clean-up. Chronically polluted El-Max district represents a very interesting site for this research topic since, due to the occurrence of strong selective pressure, most of the autochthonous bacteria should be able to cope with the environmental stressors induced by oil contamination.

## 2. Materials and Methods

### 2.1. Sites Description and Sampling

**Figure 1:**
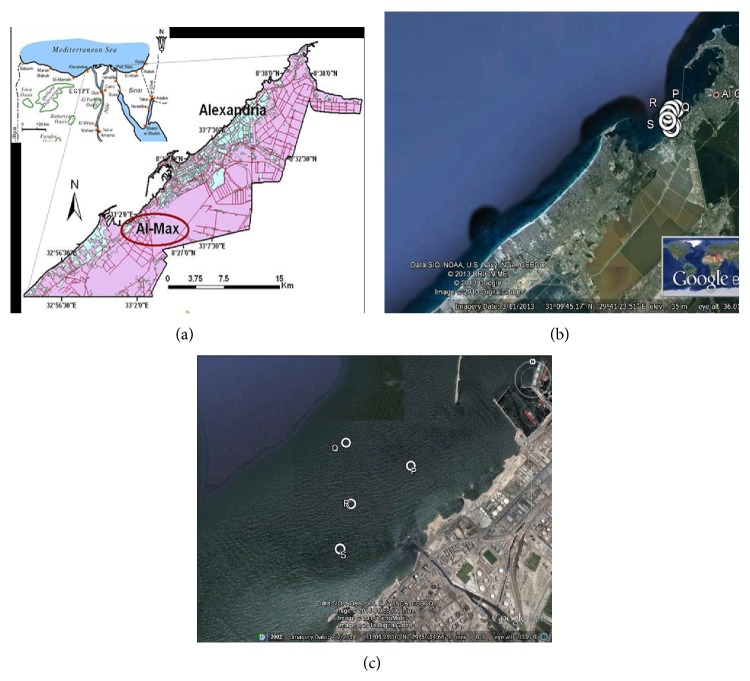
Location of the study area and sampling stations. (a) Overall area of El-Max district (Egypt) in the Mediterranean Sea and (b) satellite image of the sampling area, (c) showing the position of the four sampling sites.

*The sampling* areas are located at El-Max bay, which lies in the western side of Alexandria at longitude 29°78 E and latitude 31°13 N (Figure 1(a)). The shoreline is mainly rocky with occurrences of narrow sandy beaches. There are pronounced differences in direction and intensity of marine currents in the bay near the outlets [9, 10]. Sediment samples were collected in triplicate at depth between 3 and 16 meters, using a grab sampler, from 4 stations (Figures 1(b) and 1(c)): P (31°9′31.20′′N, 29°50′28.20′′E), Q (31°9′28.40′′N, 29°50′14.40′′E), R (31°9′18.56′′N, 29°50′5.89′′E), and S (31°9′4.89′′N, 29°50′2.49′′E). Sediment samples were packed in aluminum foil for HC analysis and in plastic bags for the rest of the physicochemical parameters. The water content, particle size, and total organic carbon were determined immediately after sampling. Sediment samples were collected using sterile spoons and stored in sterile bags at 4°C for bacterial isolation and −20°C for molecular analyses.

### 2.2. Chemical Characterization of Sediment Samples

Phosphorus extraction was performed according to Aspila et al. [15]. Total phosphorus was extracted by ashing the sample at 550°C for 2.5 h and subsequent shaking with 1 N HCl for 16 hours while the inorganic phosphorus was extracted by shaking the oven-dried sediments (110°C) with 1 N HCl for 16 hours. Phosphorus determination in the two extracts was made according to the method of Murphy and Riley [16]. Organic phosphorus was calculated subtracting the value of the inorganic phosphorous from the total phosphorus.

Total nitrogen content in the sediment samples was determined by using Kjeldahl apparatus (Raypa, model: DNP–1500, R. Espinar S.L., Barcelona, Spanish) according to standard method [17].

The total organic content (TOC) was determined by the loss-on-combustion technique after removal of carbonate with dilute (IN) HCl; a portion of sediments was weighed into a porcelain crucible and ignited in a muffle furnace at 550°C for two hours. The crucible was cooled in a desiccator and reweighed and the total organic content (TOC) was calculated as the weight loss in percentage [18]. The analysis of total pesticides and polychlorinated biphenyls (PCBs) was performed as previously described [19–21].

The presence and abundance of different n-alkanes were estimated by chromatographic techniques. The n-alkane concentration was analyzed by Agilent 7890, USA. A HP-5 capillary chromatographic column (30 m × 0.32 mm I.D.) and a capillary column (30 m × 0.25 mm I.D.) were used for GC-FID and GC-MS analyses, respectively. Nitrogen was the carrier gas with 3 mL/min. Injector and detector temperature were maintained at 300°C and 320°C, respectively. The identification of n-paraffin peaks was established using a reference mixture of n-paraffin of known composition.

To determine the total content of heavy metals (copper (Cu), iron (Fe), zinc (Zn), chromium (Cr), nickel (Ni), cadmium (Cd), cobalt (Co), and lead (Pb)) and arsenic (As) in sediments, samples (0.1 g) were HNO_3_/HClO_4_ (4 : 1, v/v) digested in a microwave oven (CEM, MARS5). After digestion, the volume of each sample was adjusted to 20 mL using deionized water. Heavy metals and arsenic content was determined by inductively coupled plasma-mass spectrometry (ICP-MS, Agilent Technologies, Santa Clara, CA, USA). Standards of heavy metals and of arsenic for concentrations ranging from 0 to 1 mg/L were prepared from multielement calibration standard-2A solution (Agilent Technologies) and from sodium arsenite solution (NaAsO_2_) (Sigma-Aldrich, St. Louis, MO, USA), respectively. For all the measures by ICP-MS an aliquot of a 2 mg/L of an internal standard solution (45Sc, 89Y, 159Tb, Agilent Technologies) was added both to samples and to a calibration curve to give a final concentration of 20 *μ*g/L. The instrument was tuned daily with a multielement tuning solution for optimized signal-to-noise ratio.

### 2.3. Metagenome Extraction and 16S rRNA Amplification

Total DNA was extracted from 0.5 g of sediment using the “Power Soil” kit (MoBio Laboratories Inc., Carlsbad, CA, USA) following the manufacturer's instructions. DNA was quantified using a NanoDrop 1000 spectrophotometer (Thermo Scientific, Waltham, MA, USA). Bacterial 16S rRNA gene fragments (~550 bp) were amplified with polymerase chain reaction (PCR) using primers 907R (3′-CCGTCAATTCCTTTGAGTTT-5′) and GC-357F (3′-CCTACGGGAGGCAGCAG-5′ with a 5′-end GC-clamp) targeting a portion of the 16S rRNA gene that includes the hypervariable V3–V5 regions [22]. PCR reactions were performed as previously described [23]. Presence and length of PCR products were checked by electrophoresis in 1% w/v agarose gel prior to denaturing gradient gel electrophoresis (DGGE) analysis.

### 2.4. Denaturing Gradient Gel Electrophoresis

PCR products (~150 ng) were loaded in a 0.5 mm polyacrylamide gel (7% (w/v) acrylamide-bisacrylamide, 37.5 : 1) containing 43 to 56% urea-formamide denaturing gradient (100% corresponds to 7 M urea and 40% (v/v) formamide). The gels were run for 16 h at 60°C by applying a constant voltage of 90 V in 1X Tris-acetate-EDTA (TAE) buffer. After electrophoresis, the gels were stained for 30 min in 1X TAE buffer containing 1X SYBR Green (Molecular Probes, Leiden, Netherlands) according to manufacturer's instructions and rinsed twice for 10 min with distilled water. Gels images were captured using a Gel Doc 2000 apparatus (Bio-Rad, Milan, Italy). The band patterns of the DGGE gel were analysed using Image J software (available for free download at http://rsb.info.nih.gov/ij/). A Principal Coordinates Analysis (PCO) was performed using PRIMER v. 6.1 [24]. DGGE bands were excided from the gels with a sterile scalpel and eluted in 50 *μ*L of sterile Milli-Q water at 37°C for 4 h. The eluted DNA was amplified by PCR using primers 357F and 907R and positive amplifications were sequenced by Macrogen Inc., Korea.

### 2.5. PCR Amplification of Functional Genes

The presence of* alkB* gene, encoding for alkane hydroxylase, in the metagenome extracted from the sediments was assessed using the primers D-alkF (5′-GCICAYGARYTIGGICAYAAR-3′) and D-alkR (5′-GCRTGRTGRTCISWRTG-3′) [25]. PCR amplification was performed in 50 *μ*L reaction containing 1X buffer, 2 mM MgCl_2_, 0.12 mM of dNTPs mixture, 1 *μ*M of each primer, 5% DMSO, 1.5 U Taq polymerase, and 10 ng of template, applying the following thermic protocol: 94°C for 4′, followed by 30 cycles of 94°C for 45′′, 55°C for 1′, and 72°C for 1′, and a final extension at 72°C for 10 min.

Primers nccA-F (5′-ACGCCGGACATCACGAACAAG-3′) and nccA-R (5′-CCAGCGCACCGAGACTCATCA-3′) were used as previously reported [12] to amplify the* nccA* gene that encode for nickel-cobalt-cadmium efflux pump. Primers dacr5F (5′-TGATCTGGGTCATGATCTTCCCVATGMTGVT-3′) and dacr4R (5′-CGGCCACGGCCAGYTCRAARAARTT-3′) were used for amplification of arsenite efflux pump (*ACR3(2)*) according to Achour et al. [13]. Primers Phn321F (5′-TTCTCGGTCGGG ACTTTCCAA-3′) and Phn671R (5′-GGCAACCAGATCTGTCATG-3′) were used for amplification of* phnA1* gene coding for 3,4-phenanthrene dioxygenase, according to Cavalca et al. [14]. PCR reactions were performed in a final volume of 25 *μ*L containing 1X buffer, 1.75 mM MgCl_2_, 0.2 mM of dNTPs mixture, 0.4 *μ*M of each primer, 1.5 U Taq polymerase, and 10 ng of total DNA.

### 2.6. Bacteria Isolation and Identification

Bacteria were enriched and isolated using two different marine mineral media (artificial seawater (ASW) and ONR7a) [3, 26] supplemented with 1% crude oil (see [27] for composition details). Enrichment vials were incubated at 30°C under agitation until turbidity was observed before proceeding with isolation. Twenty-five bacterial isolates have been obtained in pure cultures from both media. DNA extraction was performed on each isolate by boiling lysis or using Thermo Scientific GeneJET Genomic DNA Purification Kit. The amplification of the bacterial 16S rRNA gene was performed using the universal primers 27F (3′-AGAGTTTGATCMTGGCTCAG-5′) and 1492R (3′-CTACGGCTACCTTGTTACGA-5′). The PCR amplification conditions and thermal protocol were set up as previously described [23] providing a PCR amplicon of approximately 1400 bp.

### 2.7. Nucleotide Sequence Analyses and Accession Numbers

Nucleotide sequences were edited in Chromas Lite 2.01 (http://www.technelysium.com.au) and subjected to BLAST search (http://blast.ncbi.nlm.nih.gov/Blast.cgi). The partial 16S rRNA gene sequences obtained from the bacterial isolates have been deposited in the GenBank and ENA (European Nucleotide Archive) databases and the related accession number is reported in Table 6. The sequences obtained from the excised DGGE bands are available at ENA under the accession numbers LN610485–LN610498.

### 2.8. Evaluation of Metabolic Traits, Biofilm, and Biosurfactant Production within the Bacteria Collection

The potential ability to produce biosurfactant has been assessed within the bacteria collection using different assays aimed at determining the surface tension reduction, hemolytic activity, and cell hydrophobicity as previously described [3].

Surface tension of cell-free ASW medium was measured after 7 days of enrichment by tensiometer (model TD 1 LAUD, Germany) using the ring method, at room temperature [28]. The results are reported in Table 7 as mean and standard deviation of three measurements. The reduction of surface tension was determined by comparing the surface tension of the noninoculated medium (65.66 ± 4 mN/m) with the cell-free medium obtained after the incubation of tested bacteria. Biofilm formation was evaluated by using 96-well microtiter plate according to published protocols [3, 29]. The inoculated plates were incubated for 48 h at 30°C. After incubation, quantitative analysis of biofilm production was performed by measuring the optical densities (OD) at 570 nm of stained adherent bacterial films using a microtiter-plate reader (Tecan Sunrise Remote, Austria). Each assay was performed in triplicate. The noninoculated medium was used as negative control to determine background OD. The average OD values were calculated for all tested strains and negative controls. All the OD measurements were normalized against the negative control for each microtiter plate separately. The isolates were considered biofilm producers if they showed an OD value of 0.12. If the OD exceeded 0.240, they were classified as strongly adherent. Strains displaying OD values greater than 0.12 but less than 0.240 were classified as weakly adherent [30, 31]. Cell hydrophobicity was determined for bacterial isolates according to previously established protocols [32, 33]. Bacterial cells were enriched in 20 mL ASW medium supplemented with crude oil (1% w/v) and incubated for 7 days in an orbital shaker at 30°C. Cells were harvested at the early log phase, washed with phosphate buffer, and resuspended to get an initial OD600 measure comprised between 0.4 and 0.6. Cell suspension (3 mL) and crude oil (150 *μ*L) were mixed using a vortex for 120 seconds, the phases separated for 15 min, and the aqueous phase was carefully removed with a Pasteur pipette and transferred to a cuvette to measure the OD600. The decrease in the turbidity of the aqueous phase correlates with the hydrophobicity of the cells [34]. The percentage of cells bound to the hydrophobic phase (*H*) is calculated by the following formula *H* = (1 − *A*/*A*_0_) − 100%, where *A*_0_ is the absorbance of the bacterial suspension without hydrophobic phase added and *A* is the absorbance after mixing with hydrophobic phase.

The capability of each of the bacteria to utilize different HC molecules (xylene, octane, pyrene, dibenzothiophene, phenanthrene, and naphthalene) as sole carbon source was tested in ASW agar medium with a final concentration of 25 mg/L of the different HC. Xylene, octane, and naphthalene were added in the inner side of the lids of Petri dishes and incubated upside down to allow the upwards diffusion of the HC through the medium, whereas the other HCs were spread on the medium surface. The plates were incubated at 30°C for two weeks: if colonies could be detected on the plates, the ability to grow in presence of a certain compound was considered positive [3].

### 2.9. Statistical Analyses

Significant differences in the bacterial community composition were analyzed by permutational analysis of variance (PERMANOVA, [35]) considering the sampling stations and the type of sediment as an orthogonal fixed factor. All the statistical tests were performed by PRIMER v. 6.1 [24], PERMANOVA+ for PRIMER routines [36]. To assess the significance correlation between environmental data with the bacterial community composition obtained by DGGE, a Mantel test was performed (R packageade4, mantel.rtest, 999 iterations [37]).

Furthermore, distance-based multivariate analysis for a linear model (DistLM [38]) was carried out to determine the significant environmental variables explaining the observed similarity among the samples. The Akaike information criterion (AIC) was used to select the significant predictor variables. The contribution of each environmental variable was assessed using a “sequential test” to evaluate the cumulative effect of the environmental variables explaining biotic similarity.

## 3. Results and Discussion

### 3.1. Physicochemical Analyses Indicate High Level of Pollution in El-Max District Sediments

**Figure 2:**
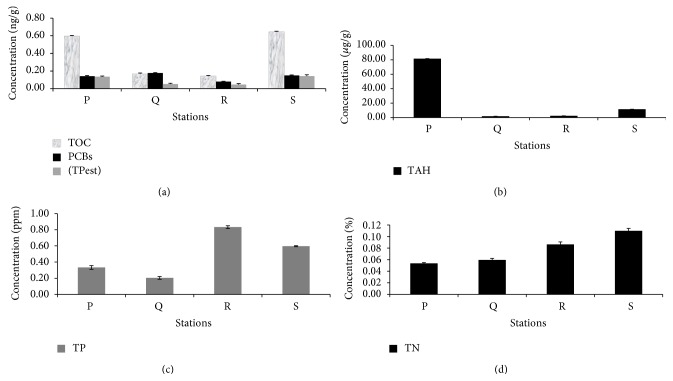
Chemical characterization of the sediments. Concentration in the sediment of (a) total nitrogen (TN); (b) total phosphorous (TP); (c) total organic carbon (TOC), total pesticides (TPest), and polychlorinated biphenyls (PCBs); (d) total aromatic hydrocarbons.

**Figure 3:**
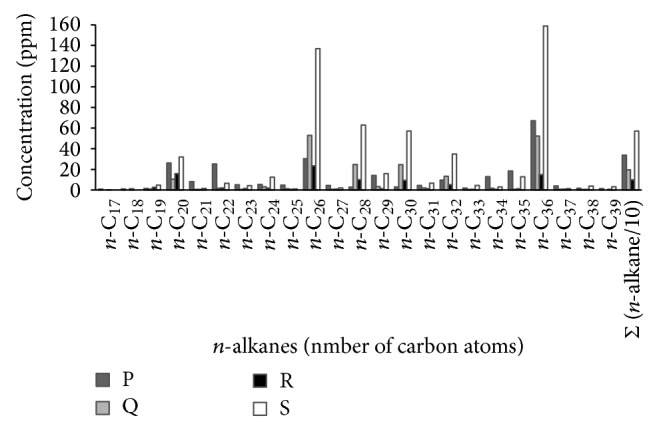
Concentration of n-alkanes in El-Max district sediments.

Physical analyses showed that the sediments collected from the stations P, Q, and R are mainly composed of sand (85.82–95.62%) while the sediment of station S displayed a different composition, containing approximately the same percentage of sand (39.41%) and silt (34.39%) and a higher proportion of clay (26.20%) compared to the rest of the stations (0–5.49%) (Table 1). Grain size measurements of superficial sediment revealed that stations P and Q contained coarse sand whereas station R displayed medium size sand (Table 1). The highest water content percentage was detected in station S (35%) which contains a fine silty sediment type (Table 1). Such differences in the water content and grain size are known to influence the solubility of elements and nutrients in marine sediments, ultimately affecting the distribution of metals and other pollutants that preferentially bind to fine particles [39], determining as a consequence that the four stations analyzed constitute different environmental niches. All the stations showed total nitrogen content below 0.2% w/v (Figure 2(a)). Stations R and S showed a high content of total phosphorous with 0.83 and 0.59 ppm, respectively (Figure 2(b)). In the case of station R, which showed the highest concentration, this could be due to the close presence of the agricultural drain El-Umum. As shown in Figure 2(c), sediments of the stations P and S displayed the highest concentrations of total organic carbon (0.56 and 0.634 ng/g, resp.) and total pesticides (0.1362 and 0.1452 ng/g, resp.). Moreover, sediments P and S contained high concentrations of PCBs (Figure 2(c)), whose highest concentration was recorded in sediments of station Q (0.17 ng/g). The assessment of total polycyclic aromatic hydrocarbons (PAH) concentration in the sediment was performed by measuring the content of 16 different aromatic hydrocarbons and it indicated that station P has the highest PAH level (Figure 2(d)). Stations P and S, containing 81.6 and 11.6 *μ*g/g of PAH, are active fishing area characterized by PAH concentration higher than the maximum indicated by the quality standards for marine water [40] and allowed by the EU (0.20 *μ*gL^−1^) and US (ΣPAH = 0.030 *μ*gL^−1^) Environmental Quality Criteria for protection of human consumers of aquatic life [41]. GC analysis of HC compounds in the analyzed stations (Figure 3) revealed that the dominant n-alkanes were* n*-C_20_ (eicosane),* n*-C_26_ (hexacosane)_,_ *n*-C_28_ (octacosane),* n*-C_30_ (tricontane), and* n*-C_36_ (hexatriacontane). Sediment collected at station P contained the highest concentration of n-alkane of* n*-C_22_, while the sediment of station S showed the highest concentrations of long chain n-alkanes of* n*-C_26_,* n*-C_28_, and* n*-C_30_. Overall, the sediment collected at station S showed the highest total n-alkanes content (Figure 3).

Heavy metal and metalloids content in sediments was evaluated by measuring 8 different metals (Cu, Fe, Zn, Cr, Ni, Cd, Co, and Pb) and As. It is noted that station S has the highest metal and As content while station P showed the lowest content (Table 2). Unlike metal concentrations in surface water, where many countries adopted clear and unambiguous guidelines (i.e., [42, 43]), there are no accepted international or local standards of metal levels in marine sediments. Only few countries (i.e., Netherlands and Canada) have a long-standing legislative tradition developing criteria and regulations for sediment quality [44], while Egypt as the majority of the countries has not enforced any environmental protection laws or the existing legislation is not clear. Thus, the metals and arsenic levels assessed in this study were compared to the EU intervention limits imposed by the law for soil and subsoil of residential or industrial areas and to literature data. In all the sediments, metals content was below the threshold concentration of European Union Standard [45] and, in particular, Ni, Pb, and Cd were retrieved at a similar level present in rural soils of many countries [46]. In addition, arsenic content was present at a comparable level to the uncontaminated soils [47].

### 3.2. El-Max District Polluted Sediments Host Complex Bacterial Communities Whose Diversity Is Driven by Physicochemical Parameters

**Figure 4:**
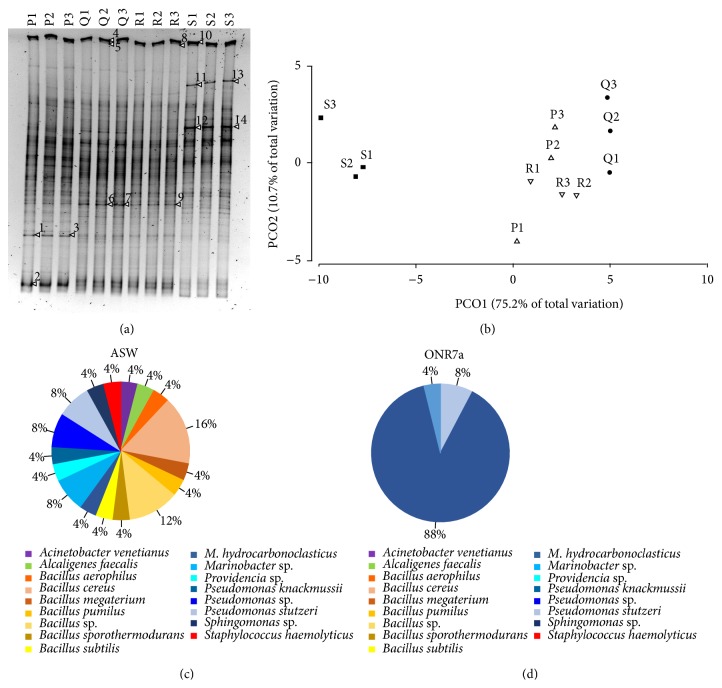
Cultivation dependent and independent analyses of the bacterial communities at El-Max district. (a) DGGE analysis performed of the 16S rRNA on the sediment metagenome. The numbers in the name of the samples represented the three analyzed replicates. Bands numbered have been excised from the gel and their DNA content has been sequenced (results are reported in Table 5). (b) Principal component analysis based on the DGGE profiles of the 16S rRNA gene in the sediments. (c) Identification and relative abundance of the bacteria isolated on ONR7a medium. (d) Identification and relative abundance of the bacteria isolated on ASW medium.

Denaturing Gradient Gel Electrophoresis (DGGE) was applied to the metagenome extracted from the sediments to provide a snapshot of the bacterial communities' structure. From each station, total sediment DNA was extracted and analyzed by DGGE. Fingerprinting performed in triplicate demonstrated the reliability of the obtained DGGE profiles (Figure 4(a)). DGGE patterns showed the occurrence of complex bacterial communities in all the analyzed sediments (Figure 4(a)) indicating that the pollution level did not affect the taxonomic diversity of bacterial communities. A positive correlation between the environmental data available for the analyzed sediments and the detected DGGE pattern was indicated by the Mantel test (*r* = 0.597; *P* < 0.05), revealing that the physical parameters, together with the measured nutrients and pollutants concentration, are the main drivers of the overall composition of the bacterial communities. The DGGE patterns of the sediments P, Q, and R appeared to be similar whereas differences could be observed with sediment S, concerning the presence of peculiar bands as well as differential abundance of some ubiquitous bands (Figure 4(a)). Principal Coordinates Analysis (PCO) of the DGGE fingerprints confirmed the observed differences showing a sharp clustering of sediments S1-2-3 separately from the other sediments (Figure 4(b)). Based on PCO1 (explaining 75.2% of the total variation) the bacterial community of the samples collected at station Q were also different from the sediments P and R (Figure 4(b)). Statistical analysis supported the PCO indications, confirming that both the bacterial community dwelling sediments S and Q were significantly different from those collected at stations P and R (see Table 3(a) for pairwise comparison). Moreover, PERMANOVA showed that the structure of sediments' bacterial community was influenced by the type of sediment (PERMANOVA, df = 2, *F* = 12.7, *P* = 0.0001), distinguishing sandy (P, Q, and R) and silty (S) sediments as statistically different (see Table 3(b) for pairwise comparison).

Physical and chemical parameters measured at the investigated stations were analyzed to assess their influence on the structure of the bacterial communities (Table 4). The sequential test showed that sand and clay percentage in the sediments are the statistically significant physical parameters involved in shaping the bacterial communities (Table 4(a)). Furthermore, DistLM analysis was performed on chemical data considering separately the metal concentration and the other available chemical parameters. The sequential tests showed that, among metals, Cu, Fe, and Zn concentration are the drivers of the bacterial community structure in the sediments (Table 4(b)) and that total organic carbon (TOC) and PCBs concentration were the other statistically significant parameters involved in the selection and assemblage of bacterial populations (Table 4(c)). Our data are in agreement with a recent study, which indicated both sediment particle size and the concentration of metals, including Fe and Zn, as pivotal factors in shaping the sediment's bacterial community [48].

Aiming to identify the dominant taxa associated with the PCR-DGGE profiles several DGGE bands were excised from the gel. The successful sequencing of partial 16S rRNA could be obtained only for 14 bands (Figure 4(a)), pointing out the presence of bacteria typical of marine ecosystems and characterized by low identity percentage with any known sequence in the public databases (Table 5). According to DGGE band sequencing, the main phylum associated with El-Max district sediment was represented by Bacteroidetes, while Actinobacteria, Acidobacteria, and Spirochaetes were retrieved in lower abundance (Table 5). The phylum Bacteroidetes was previously indicated among the main actors involved in PAH degradation in river sediment based on DGGE analyses [49] and could possibly play a role in marine sediments. The phyla Actinobacteria, Acidobacteria, and Bacteroidetes were also detected by high-throughput sequencing in estuarine sediments [48] while Spirochaetes were identified within the metabolically active bacterial communities in microcosms established using chronically polluted estuarine sediments [50].

The presence of putative HC-oxidizers in El-Max district sediments was, moreover, demonstrated by the amplification of the* alkB* gene, codifying the alkane monooxygenase enzyme, from all the sediment metagenomes (data not shown).

### 3.3. Phylogenetically Different Hydrocarbonoclastic Bacterial Isolates Were Obtained Using Different Cultivation Media

Besides molecular analysis, a cultivation approach was applied to obtain and characterize bacterial isolates according to the biotechnological potential for bioremediation applications. Twenty-five bacteria were isolated from the four stations on ASW medium supplemented with crude oil as the sole carbon source. This collection included Bacilli (13), Betaproteobacteria (1), and Gammaproteobacteria (11) divided into several families and genera (Table 6), with a high Shannon-Weaver index (2.69), calculated from the number of individuals per genus. The collection included bacterial genera widely studied for their ability to degrade oil hydrocarbons, such as* Acinetobacter venetianus *[51],* Pseudomonas stutzeri* [52], and* Marinobacter hydrocarbonoclasticus *[53] (Table 6, Figure 4(c)). Bacteria belonging to the genera* Acinetobacter, Pseudomonas, *and* Marinobacter *were isolated from a variety of oil contaminated sites around the world. Such environments included coastal oil-polluted site in Tunisia [54], intertidal sand affected by oil pollution after the Prestige spill [55], the Gulf of Mexico beach sand [56], and deep hypersaline anoxic basins [57]. Kostka and coauthors [56] recently proposed that Gram-positive bacteria like those of the genus* Bacillus*, representing the 48% of the bacteria we isolated on ASW medium, could be used as sentinel for the later stages of oil degradation, when PAH dominate the composition of the residual oil. Accordingly, other authors [58–60] previously reported the presence of hydrocarbon-degrading* Bacillus* strains from marine sediments and seawater. The fraction of El-Max sediment bacteria cultivable on ASW medium included representatives of the genera* Alcaligenes*,* Sphingomonas,* and* Providencia *(Table 6, Figure 4(d)), the latter showing high potential for the bioremediation of heavy metals [61, 62], which are abundantly present in the samples analyzed in this study. Marine bacteria able to resist high mercury concentrations and able to detoxify cadmium and lead were described also within the species* Bacillus pumilus* and* Alcaligenes faecalis *[63], present in our collection (Table 6).* A. faecalis* can perform phenanthrene degradation [64] and was also previously detected by DGGE analysis in weathered fuel enrichment established after the Prestige oil spill [65]. The ability of* Sphingomonas* spp. isolates to degrade a wide range of xenobiotics has been reported and their remediating capability has been assigned to a large plasmid harboring the genes codifying degrading enzymes [64].

Sediment from station S, which hosts a peculiar bacterial community according to the DGGE analysis, was used to perform second enrichment on ONR7a medium, adding crude oil as the sole carbon source. A second collection of twenty-five bacteria was obtained from the ONR7a enrichment, leading to the selection of three different species, thus showing a lower diversity compared to the ASW collection (Shannon-Weaver index: 0.44). All the bacteria isolated on ONR7a medium belonged to the known hydrocarbon-degrading species* Pseudomonas stutzeri* (2),* Marinobacter adhaerens* (1), and* Marinobacter hydrocarbonoclasticus *(22) (Table 6). It is worthy to note how much our perception of the cultivable fraction of oil-degrading bacteria in a certain environment can vary simply changing the medium applied for cultivation purposes. In fact, ONR7a medium exclusively selected Gammaproteobacteria from El-Max district polluted sediments, mainly represented by the well-known metabolic versatile* Marinobacter hydrocarbonoclasticus*. The cultivation approach based on the use of two different media provided a wider perspective on the cultivable fraction of the bacterial community present in the investigated sediments. Such strategy permitted identifying both (i) specialist and versatile bacterial species involved in the first stages of the degradation of aliphatic and aromatic hydrocarbons (i.e., Gammaproteobacteria) and (ii) bacterial species previously indicated as key players in the successional stages of the degradation process (i.e.,* Bacillus*).

### 3.4. Hydrocarbonoclastic Bacteria of El-Max District Possessed Bioremediation Potential Traits

We screened the bacterial collection for the ability to grow on single hydrocarbon molecules as sole carbon source, showing that a variable percentage of the isolates were able to grow on the different tested HC (Table 7). A lower percentage of the strains were able to grow using pyrene (7%) and phenanthrene (8%) and naphthalene (9.5%). A higher portion of the collection could grow using dibenzothiophene (DBT, 11%), octane (15.5%), and xylene (36%). Both the collections obtained on ASW and ONR7a media included similar amounts of strains able to use all the tested hydrocarbons, with the exception of DBT and octane degrading bacteria that were mainly isolated on ASW and ONR7a medium, respectively. The ability to grow using all the supplied HC molecules in minimal medium was recorded exclusively in few strains belonging to the species* Marinobacter hydrocarbonoclasticus* (Table 7), confirming the adaptable metabolisms of this species often isolated in marine contaminated environments. The low number of bacteria capable of utilizing all the tested substrates corroborates the reports of several studies that indicate the need of microbial consortia to degrade complex mixtures of hydrocarbons, such as crude oil, in soil [66], fresh water [67], and marine environments [4].

To widen the characterization of the HC-degrading bacteria isolated from El-Max district we performed PCR assays looking for functional genes codifying the 3,4-phenanthrene dioxygenase enzyme (*phnA*) and genes related to metal and metalloid detoxification systems, like the efflux pumps for arsenite (*ACR3(2)*) and for different heavy metals (*nccA*). Despite the fact that the sediments were not highly contaminated by metals and arsenic, isolates possessing arsenic and metals resistance genes were retrieved, confirming that bacteria capable of detoxification mechanism are widespread and their presence is not strictly related to metal and arsenic level [13, 68].* ACR3(2)*,* nccA,* and* phnA* genes were successfully amplified in 14, 10, and 4 bacterial strains isolated on ONR7a medium, respectively. On the contrary, the amplification of these genes was unsuccessful for all the bacteria isolated on ASW medium, with the exception of the strain* Alcaligenes faecalis* SCP3, which resulted positive for* nccA* gene amplification and belongs to species previously described as Cd and Pb detoxifying [63]. Overall, apart from strain* A. faecalis* SCP3, all the bacteria positive for* ACR3(2)*,* nccA,* and* phnA* genes amplification belong to the species* M. hydrocarbonoclasticus*. Only one out of the 4 strains harboring the* phnA* gene was able to grow on phenanthrene as the sole carbon source in the tested conditions. On the other hand, those that could grow on this HC failed to give positive amplification, probably due to mismatches between the tested primers and gene sequence [69].

The biotechnological potential of the strains inhabiting oil-polluted ecosystems does not rely exclusively on their ability to degrade a certain HC mixture but it includes additional features. Different microorganisms were shown to possess multiple adaptations to facilitate oil degradation procedures, such as the synthesis of biosurfactants or emulsifiers and biofilm formation [70, 71], processes that enhance the bacterial adhesion to hydrocarbons, increasing their solubility and thus promoting their degradation [72, 73]. Biosurfactants, in particular, reduce the surface tension at the interface of immiscible fluids, increasing the surface area of insoluble compounds like oil and water, which leads to increased bioavailability and subsequent biodegradation of the hydrocarbons [4, 74, 75]. In this study, we applied several methods to assess the ability of the isolated bacteria to produce biosurfactant molecules. One of the simplest methods used for screening the production of some types of biosurfactants is the blood haemolysis method [11]. In our study, 20 isolates (40% of the collection) showed haemolytic activity on blood agar media (Table 7). They belong to the* Marinobacter *(11),* Bacillus *(5),* Pseudomonas* (3), and* Acinetobacter* (1) genera. The reduction of the surface tension (ST) of the medium, resulting from the emulsification of crude oil by the surfactants produced by microorganism, represents an alternative method for testing the biosurfactant production [76]. All the isolates in our collections were able to reduce surface tension when compared to the noninoculated medium surface tension (65.66 ± 4 mN/m) (Table 7); in particular those isolated on the ONR7a medium (average value within the collection: 27.4 ± 10.2), namely,* M. hydrocarbonoclasticus*, demonstrated higher ST reduction compared to those belonging to the ASW collection (average value within the collection: 47.25 ± 10.36). Furthermore, bacterial adhesion to hydrocarbons (BATH) test was applied to measure the cell surface hydrophobicity, a property related to the structure and composition of cell surface [77]. The uptake mechanism of hydrophobic substrate occurs by the direct contact between the hydrocarbon and cell surface and can be thus dependent on its hydrophobicity [28, 77, 78]. The highest hydrophobicity (77.3%) was recorded for the strain* M. hydrocarbonoclasticus* SCS6 (Table 7).

Hydrocarbonoclastic bacteria have been detected in both monospecies and multispecies biofilms developing on hydrocarbons [79]. Hence, the ability to produce biofilm was also investigated (Table 7) allowing the identification of 5 strains, representing 10% of the collection, as biofilm producers. Such strains belong to the* M. hydrocarbonoclasticus, A. faecalis, B. cereus, *and* P. vermicola *species. According to the literature [30, 31] two strains were classified as weakly adherent, while two resulted in being strongly adherent (Table 7), the latter including the* M. hydrocarbonoclasticus* strain S1-21. A proteomic study realized on* M. hydrocarbonoclasticus* previously showed a differential protein expression for biofilm attached and detached cells, displaying the ability of recently detached cells to reinitiate the formation of a new biofilm at the hexadecane-water interface [79], a trait that might confer competitive advantage for hydrocarbon uptakes in the environment. The two* M. hydrocarbonoclasticus* isolates (S1-4 and S1-21) positive for biofilm formation were also able to grow using the entire set of hydrocarbons tested in this study, further demonstrating the high potential of this species for marine oil remediation.

## 4. Conclusions

Thisstudy represents the first holistic microbiological investigation of biodiversity occurring at El-Max district sediments taking advantage of both molecular and cultivation techniques. The adopted molecular approach, coupled with statistical analyses, clarified that a significant correlation exists between biotic and abiotic data in the polluted ecosystems, allowing identifying (i) sand and clay composition, (ii) TOC and PCBs, and (iii) the concentration of different heavy metals (Cu, Fe, and Zn) as the driving forces shaping the structure of the bacterial microbiome. The establishment of a bacterial collection exploiting different growth media permitted isolating species described for their pivotal role in the different successional stages of oil hydrocarbons' biodegradation, such as the highly abundant classes Gammaproteobacteria and Bacilli. Most of the isolates, belonging to different genera, showed one or more metabolic traits of interest for bioremediation purposes (e.g., the capability to grow on single hydrocarbon molecules, presence of genes involved in detoxification systems, and traits related to the production of biosurfactants). Our investigation contributed to filling the gap of knowledge on the microbial diversity of Southern Mediterranean Sea sites, shedding light on the potential of the contaminated sediments of El-Max district as a reservoir of microbial resources selected (and adapted) by the peculiar environmental conditions of the site and possibly exploitable for future* in situ* intervention to combat pollution.

## Figures and Tables

**Table 1:** Physical characteristics of El-Max district sediments samples.

Station	% sand	% silt	% clay	Mean size (phi)	Type of sediment	Water content %	Porosity %
P	95.18	4.82	0.00	0.11	Coarse sand	15.00	6.90
Q	85.82	8.7	5.49	0.49	Coarse sand	20.00	9.79
R	95.62	3.04	1.34	1.51	Medium sand	28.00	15.02
S	39.41	34.39	26.20	4.84	Coarse silt	35.00	20.22

**Table 2:** Total heavy metal and arsenic content in El-Max district sediments.

	Cu (mg/kg)	Fe (g/kg)	Zn (mg/kg)	Cr (mg/kg)	Ni (mg/kg)	Cd (mg/kg)	Co (mg/kg)	Pb (mg/kg)	As (mg/kg)
P	22.63 ± 4.09	4.389 ± 0.21	45.77 ± 10.42	19.21 ± 1.27	7.83 ± 2.12	1.19 ± 1.93	1.58 ± 0.03	19.27 ± 4.87	3.31 ± 0.57
Q	65.98 ± 11.1	11.16 ± 1.69	142.97 ± 17.16	78.35 ± 10.19	15.93 ± 2.95	0.25 ± 0.14	3.71 ± 0.69	44.15 ± 0.44	4.90 ± 0.84
R	72.79 ± 1.66	11.23 ± 0.75	142.80 ± 11.16	86.6 ± .3.66	19.18 ± 0.74	0.28 ± 0.05	4.11 ± 0.31	45.57 ± 2.47	5.17 ± 0.93
S	118.15 ± 12.14	11.66 ± 0.93	247.71 ± 22.59	105.08 ± 7.69	26.37 ± 2.08	0.58 ± 0.11	4.45 ± 0.54	59.40 ± 5.01	7.06 ± 1.25

**(a):** 

Groups	*t*	*P*
**P, Q**	**2,1697**	**0,0385**
P, R	1,6055	0,1077
**P, S**	**3,7151**	**0,0044**
**Q, R**	**2,6056**	**0,0146**
**Q, S**	**5,9167**	**0,0009**
**R, S**	**5,1805**	**0,002**

**(b):** 

Groups	*t*	*P*
Coarse sand, medium sand	1,2488	0,2166
**Coarse sand, coarse silt**	**4,3187**	**0,0006**
**Medium sand, coarse silt**	**5,1805**	**0,0021**

**(a):** 

Sequential tests						
Variable	AIC	*F*	*P*	Prop.	Cumul.	Res.df
**+% sand**	**35,267**	**16,445**	**0,0036**	**0,62186**	**0,62186**	**10**
**+% silt**	**34,458**	**2,3747**	**0,0738**	**0,078943**	**0,70081**	**9**
**+% clay**	**28,526**	**7,493**	**0,0008**	**0,1447**	**0,84551**	**8**
Mean size	28,526	No test		−1,60*E* − 14	0,84551	8
Water content	28,526	No test		−1,59*E* − 16	0,84551	8
Porosity	28,526	No test		1,14*E* − 15	0,84551	8

**(b):** 

Sequential tests						
Variable	AIC	*F*	*P*	Prop.	Cumul.	Res.df
**Cu**	**40,706**	**6,8084**	**0,0092**	**0,40506**	**0,40506**	**10**
**Fe**	**30**	**16,947**	**0,0009**	**0,38858**	**0,79364**	**9**
**Zn**	**28,526**	**2,686**	**0,055**	**0,051871**	**0,84551**	**8**
Cr	28,526	No test		−4,67*E* − 16	0,84551	8
Ni	28,526	No test		−6,42*E* − 16	0,84551	8
Cd	28,526	No test		7,80*E* − 16	0,84551	8
Co	28,526	No test		−2,21*E* − 16	0,84551	8
Pb	28,526	No test		5,79*E* − 16	0,84551	8
As	28,526	No test		7,27*E* − 16	0,84551	8

**(c):** 

Sequential tests						
Variable	AIC	*F*	*P*	Prop.	Cumul.	Res.df
TAH	46,138	0,68916	0,506	6,45*E* − 02	6,45*E* − 02	10
**TOC**	**31,775**	**26,19**	**0,0006**	**0,69626**	**0,76073**	**9**
**PCHs**	**28,526**	**4,3898**	**0,0099**	**8,48** **E** − 02	**0,84551**	**8**
Tpest	28,526	No test		3,40*E* − 16	0,84551	8
TN	28,526	No test		−9,57*E* − 16	0,84551	8
TP	28,526	No test		8,47*E* − 16	0,84551	8

**Table 5:** Phylogenetic identification of bacteria from sequenced DGGE bands (see Figure 4 for band correspondence). The column “Environment” reports the habitat in which the “Closest relative” sequence present in NCBI database was detected.

Band	Sample	Closest relative (Acc. No.)	Identity (%)	Phylum	Environment
1	P1	Unc. Bacterium (FR851749)	99	Actinobacteria	Coral reef sands
2	P1	Unc. Acidobacteria (JF344347)	97	Acidobacteria	Oil-polluted sediments
3	P3	Unc. Bacterium (FR851749)	99	Actinobacteria	Coral reef sands
4	Q2	Unc. Bacterium (JN453366)	95	Bacteroidetes	Hypersaline microbial mat
5	Q2	Unc. Bacterium (JN470103)	96	Bacteroidetes	Hypersaline microbial mat
6	Q2	Unc. Bacterium (JN530286)	98	Spirochaetes	Hypersaline microbial mat
7	Q3	Unc. Bacterium (JN530286)	97	Spirochaetes	Hypersaline microbial mat
8	R3	Unc. Bacterium (KC574864)	95	Bacteroidetes	—
9	R3	Unc. Bacterium (KC574864)	94	Bacteroidetes	Hypersaline microbial mat
10	S1	Unc. Bacterium (JN529047)	93	Bacteroidetes	Hypersaline microbial mat
11	S1	Unc. Bacteroidetes (AF507860)	97	Bacteroidetes	Meromictic soda lake
12	S1	Unc. Bacterium (KF268891)	99	Bacteroidetes	Marine sediment
13	S3	Unc. Bacteroidetes (AF507860)	97	Bacteroidetes	Meromictic soda lake
14	S3	Unc. Bacterium (KF268891)	99	Bacteroidetes	Marine sediment

**Table 6:** List of the bacterial strains isolated from the polluted sediments of El-Max district (Egypt) and their phylogenetic affiliation. The codes of the strains isolated from station R are indicated in italics since their identification was previously reported by the same authors [11].

Strain code	Medium	Acc. No.	Class	Family	Closest described relative	Identity (%)
SCP2	ASW	KC573500	Bacilli	Bacillaceae	*Bacillus sporothermodurans *	96
SCuQ1	ASW	KC573503			*Bacillus megaterium *	99
*SCR2* ^ a^	ASW	KC573523			*Bacillus megaterium *	98
*SCuR2* ^ a^	ASW	KC573507			*Bacillus cereus *	98
*SCR3* ^ a^	ASW	KC573505			*Bacillus cereus *	99
*SC∗R3* ^ a^	ASW	KF217252			*Bacillus cereus *	99
*SCuR5* ^ a^	ASW	KF217249			*Bacillus* sp.	99
SC∗S1	ASW	KF217253			*Bacillus pumilus *	99
SCS2	ASW	KC573509			*Bacillus cereus *	99
SCS3	ASW	KC573510			*Bacillus subtilis *	99
SCS4	ASW	KF217259			*Bacillus* sp.	99
SC∗S6	ASW	KF217254			*Bacillus aerophilus *	99
SCP1	ASW	KC573499		Staphylococcaceae	*Staphylococcus haemolyticus *	99
SC∗CuP1	ASW	KC573501	Gammaproteobacteria	Pseudomonadaceae	*Pseudomonas xanthomarina *	98
SCuQ2	ASW	KC573504		Pseudomonadaceae	*Pseudomonas stutzeri *	99
*SCuR3* ^ a^	ASW	KC573508		Pseudomonadaceae	*Pseudomonas knackmussii *	98
*SCuR4* ^ a^	ASW	KC573524		Pseudomonadaceae	*Pseudomonas stutzeri *	99
SCS1	ASW	KC573525		Pseudomonadaceae	*Pseudomonas stutzeri *	100
SC∗Q2	ASW	KC573520		Moraxellaceae	*Acinetobacter venetianus *	99
*SCR1* ^ a^	ASW	KC573522		Alteromonadaceae	*Marinobacter hydrocarbonoclasticus *	99
SC∗Q3	ASW	KC573502			*Marinobacter hydrocarbonoclasticus *	98
SCS6	ASW	KC573526			*Marinobacter hydrocarbonoclasticus *	99
*SC∗R2* ^ a^	ASW	KF217251		Enterobacteriaceae	*Providencia vermicola *	99
*SCuR1* ^ a^	ASW	KC573506		Sphingomonadaceae	*Sphingomonas* sp.	95
SCP3	ASW	KF217258	Betaproteobacteria	Alcaligenaceae	*Alcaligenes faecalis *	99
S1_1	ONR7a	LN610460	Gammaproteobacteria	Pseudomonadaceae	*Pseudomonas stutzeri *	99
S1_24	ONR7a	LN610475			*Pseudomonas stutzeri *	99
S1_4	ONR7a	LN610461		Alteromonadaceae	*Marinobacter hydrocarbonoclasticus *	99
S1_5	ONR7a	LN610462			*Marinobacter hydrocarbonoclasticus *	100
S1_7	ONR7a	LN610463			*Marinobacter hydrocarbonoclasticus *	99
S1_9	ONR7a	LN610464			*Marinobacter hydrocarbonoclasticus *	99
S1_10	ONR7a	LN610465			*Marinobacter hydrocarbonoclasticus *	99
S1_11	ONR7a	LN610466			*Marinobacter hydrocarbonoclasticus *	99
S1_12	ONR7a	LN610467			*Marinobacter hydrocarbonoclasticus *	99
S1_13	ONR7a	LN610468			*Marinobacter hydrocarbonoclasticus *	100
S1_16	ONR7a	LN610469			*Marinobacter hydrocarbonoclasticus *	99
S1_17	ONR7a	LN610470			*Marinobacter hydrocarbonoclasticus *	100
S1_20	ONR7a	LN61047			*Marinobacter hydrocarbonoclasticus *	100
S1_21	ONR7a	LN610472			*Marinobacter hydrocarbonoclasticus *	100
S1_22	ONR7a	LN610473			*Marinobacter hydrocarbonoclasticus *	99
S1_23	ONR7a	LN610474			*Marinobacter hydrocarbonoclasticus *	99
S1_26	ONR7a	LN610476			*Marinobacter hydrocarbonoclasticus *	99
S1_28	ONR7a	LN610477			*Marinobacter hydrocarbonoclasticus *	99
S1_29	ONR7a	LN610478			*Marinobacter hydrocarbonoclasticus *	99
S1_30	ONR7a	LN610479			*Marinobacter hydrocarbonoclasticus *	99
S1_31	ONR7a	LN610480			*Marinobacter hydrocarbonoclasticus *	99
S1_32	ONR7a	LN610481			*Marinobacter adhaerens *	100
S1_33	ONR7a	LN610482			*Marinobacter hydrocarbonoclasticus *	99
S1_34	ONR7a	LN610483			*Marinobacter hydrocarbonoclasticus *	99
S1_36	ONR7a	LN610484			*Marinobacter hydrocarbonoclasticus *	99

Acc. No.: accession number of the 16S rRNA sequences amplified from the isolated strains and deposited in GenBank.

**Table 7:** Screening of the bioremediation potential in the bacteria collection established from the sediment of El-Max district.

Isolate	Biofilm	b.h.	ST (mN m^−1^)	Hydro (%)	Growth on different hydrocarbons							PCR amplification of gene markers		
					Xyl	Oct	Pyr	DBT	Phe	Naph	Oil	*nccA* ^ (a)^	*ACR3*(*2*)^(b)^	*phnA* ^ (c)^
S1-1	0.023 ± 0.0071	−	39.33 ± 3.05	38 ± 2.466	+	+	+	−	+	+	+	−	+	−
S1-4	0.164 ± 0.0789	−	45.63 ± 0.55	n.d.	+	+	+	+	+	+	+	−	+	−
S1-5	0.024 ± 0.0081	−	10.42 ± 1.64	5.9 ± 1.322	−	+	−	−	−	−	+	−	−	+
S1-7	0.014 ± 0.0123	+	20.33 ± 1.52	5.1 ± 1.607	−	+	−	+	−	−	+	−	+	−
S1-9	0.0125 ± 0.0071	+	32.33 ± 1.52	20.3 ± 2.020	+	+	−	−	−	−	+	+	+	−
S1-10	0.0112 ± 0.0035	−	28.66 ± 1.52	46.2 ± 0.954	−	−	−	−	−	−	+	+	+	−
S1-11	0.041 ± 0.0111	+	18.66 ± 0.57	10.4 ± 1.527	−	+	−	−	−	−	+	+	+	−
S1-12	0.014 ± 0.0472	−	15.83 ± 0.76	40.8 ± 0.831	−	+	−	+	−	−	+	−	+	+
S1-13	0.024 ± 0.0081	+	12.96 ± 0.55	n.d.	−	+	−	−	−	+	+	−	+	−
S1-16	0.035 ± 0.0135	−	28.66 ± 0.57	21.98 ± 1.527	−	+	−	−	−	−	+	+/−	−	+
S1-17	0.013 ± 0.0086	+	29.3 ± 1.57	9.3 ± 0.5	−	+	−	+	−	−	+	+/−	+	−
S1-20	0.0145 ± 0.0092	−	25.23 ± 1.05	n.d.	+	+	+	+	+	+	+	−	−	−
S1-21	0.418 ± 0.0920	−	28.36 ± 1.28	10.4 ± 1.261	+	+	+	+	+	+	+	−	−	−
S1-22	0.0297 ± 0.0113	−	20.33 ± 0.57	n.d.	+	+	−	−	−	−	+	−	−	−
S1-23	0.009 ± 0.0054	+	27.23 ± 0.68	7.5 ± 0.550	+	+	−	−	−	−	+	−	−	−
S1-24	0.0126 ± 0.0034	−	23.66 ± 1.52	n.d.	+	+	+	+	+	+	+	+/−	−	−
S1-26	0.0525 ± 0.0147	+	29.1 ± 0.85	13.3 ± 0.941	+	+	+	−	−	+	+	−	+	−
S1-28	0.013 ± 0.0081	−	40.5 ± 1.32	16.3 ± 0.774	−	+	−	−	−	−	+	−	+	−
S1-29	0.004 ± 0.0021	+	20.63 ± 3.05	14.2 ± 1.286	−	+	−	−	+	+	+	−	−	+
S1-30	0.012 ± 0.0046	+	47.26 ± 0.57	21.3 ± 1.382	+	+	−	−	−	−	+	−	+	−
S1-31	0.012 ± 0.0058	−	20.36 ± 1.15	13.8 ± 0.694	−	+	−	−	−	−	+	−	−	−
S1-32	0.0183 ± 0.0078	−	40.26 ± 1.44	15.8 ± 0.390	+	+	−	−	−	−	+	+	−	−
S1-33	0.004 ± 0.0015	−	40.83 ± 0.76	34.7 ± 0.375	−	−	−	−	−	−	+	+/−	+	−
S1-34	0.0134 ± 0.007	−	15.16 ± 1.75	22.2 ± 2.450	−	+	−	−	−	−	+	−	+	−
S1-36	0.024 ± 0.0081	+	20.63 ± 3.05	n.d.	+	+	−	−	−	−	+	+	−	−
SCP1	0.011 ± 0.0075	−	47.16 ± 1.52	n.d.	−	−	−	−	−	−	−	−	−	−
SCP2	0.028 ± 0.0053	+	51.25 ± 0.77	11.3 ± 1.734	−	−	+	+	+	+	+	−	−	−
SCP3	0.417 ± 0.0156	−	48.30 ± 1.34	31.6 ± 1.443	+	+	−	+	+	+	−	+	−	−
SCR1^a^	0.046 ± 0.0122	+	53.50 ± 1.25	77.3 ± 2.508	n.d.	−	−	−	−	−	+	−	−	−
SCR2^a^	0.040 ± 0.0083	+	46.67 ± 0.84	26.8 ± 1.527	−	−	−	−	+	−	−	−	−	−
SCR3^a^	0.030 ± 0.0083	−	57.64 ± 0.50	23.5 ± 1.702	−	−	+	−	+	+	−	−	−	−
SCS1	0.037 ± 0.0132	−	52.52 ± 0.77	10.2 ± 1.297	−	−	−	−	−	+	−	−	−	−
SCS2	0.037 ± 0.0132	−	22.90 ± 1.48	23.6 ± 1.950	+	−	+	+	+	+	−	−	−	−
SCS3	0.065 ± 0.0140	+	41.56 ± 1.05	n.d.	+	+	−	+	−	−	++	−	−	−
SCS4	0.075 ± 0.0018	+	46.20 ± 1.00	2.7 ± 1.322	−	−	+	+	−	−	−	−	−	−
SCS6	0.032 ± 0.0016	−	44.35 ± 0.70	n.d.	−	+	+	+	n.d.	n.d.	+	−	−	−
SCuP1	0.022 ± 0.0106	+	50.95 ± 1.52	46.4 ± 2.466	−	−	−	+	+	+	+	−	−	−
SCuQ1	0.081 ± 0.0240	−	52.30 ± 1.07	0.96 ± 0.076	−	−	−		+	−	+	−	−	−
SCuQ2	0.007 ± 0.0097	−	59.82 ± 0.53	28.8 ± 1.258	−	−	−	−	−	−	−	−	−	−
SCuR1^a^	0.049 ± 0.0131	−	59.70 ± 1.36	7.1 ± 1.294	+	+	−	+	+	+	−	−	−	−
SCuR2^a^	0.003 ± 0.0081	−	50.09 ± 1.51	6.7 ± 0.475	−	−	−	−	−	−	−	−	−	−
SCuR3^a^	0.086 ± 0.0116	+	59.40 ± 0.72	8.7 ± 1.189	−	+	+	+	+	+	−	−	−	−
SCuR4^a^	0.051 ± 0.0116	+	35.87 ± 0.93	29.1 ± 1.322	+	+	+	+	−	−	+	−	−	−
SCuR5^a^	0.022 ± 0.0097	+	38.30 ± 1.07	22.7 ± 1.875	−	+	+	+	−	+	++	−	−	−
SC∗Q2	0.032 ± 0.0142	+	47.84 ± 1.11	9 ± 0.304	−	−	−	+	−	+	−	−	−	−
SC∗Q3	0.026 ± 0.005	−	48.71 ± 2.23	3 ± 0.132	n.d.	−	−	+	−	−	−	−	−	−
SC∗R2^a^	0.177 ± 0.0512	−	55.60 ± 1.92	6.6 ± 0.25	+	+	−	+	+	+	−	−	−	−
SC∗R3^a^	0.366 ± 0.0361	+	42.6 ± 0.808	n.d.	n.d.	n.d.	−	+	−	−	n.d.	−	−	−
SC∗S1	0.067 ± 0.0272	−	16.88 ± 0.76	36.6 ± 2.432	−	−	−	−	+	+	++	−	−	−
SC∗S6	0.012 ± 0.0083	−	51.25 ± 1.86	18.3 ± 0.125	−	−	−	−	−	−	−	−	−	−

+: growth; −: no growth; n.d.: not detected.

Xyl: xylene; Oct: octane; Pyr: pyrene; DBT: dibenzothiophene; Phe: phenanthrene; Naph: naphthalene; oil: crude oil.

ST: surface tension; Hydro: hydrophobicity ratio (%); b.h.: blood haemolysis; biofilm: biofilm formation.

^(a)^Ni, Co, and Cd efflux pump, amplified with primer nccA [12]; ^(b)^arsenite efflux pump [13]; ^(c)^3,4-phenanthrene dioxygenase large subunit amplified with primer Phn321F/P671R [14].
